# Antibiotic Susceptibility Profiling of Human Pathogenic *Staphylococcus aureus* Strains Using Whole Genome Sequencing and Genome-Scale Annotation Approaches

**DOI:** 10.3390/microorganisms11051124

**Published:** 2023-04-26

**Authors:** Mejdi Snoussi, Emira Noumi, Nouha Bouali, Abdulrahman S. Bazaid, Mousa M. Alreshidi, Hisham N. Altayb, Kamel Chaieb

**Affiliations:** 1 Department of Biology, College of Science, University of Ha’il, Ha’il P.O. Box 2440, Saudi Arabia; eb.noumi@uoh.edu.sa (E.N.); n.bouali@uoh.edu.sa (N.B.); mo.alreshidi@uoh.edu.sa (M.M.A.); 2 Medical and Diagnostic Research Centre, University of Ha’il, Ha’il 55473, Saudi Arabia; 3 Department of Medical, Laboratory Science, College of Applied Medical Sciences, University of Ha’il, Ha’il 55476, Saudi Arabia; ar.bazaid@uoh.edu.sa; 4 Department of Biochemistry, Faculty of Science, King Abdulaziz University, Jeddah 21589, Saudi Arabia; hdemmahom@kau.edu.sa (H.N.A.); kalshaib@kau.edu.sa (K.C.); 5 Center of Artificial Intelligence in Precision Medicines, King Abdulaziz University, Jeddah 21589, Saudi Arabia; 6 Laboratory of Analysis, Treatment and Valorization of Pollutants of the Environmental and Products, Faculty of Pharmacy, University of Monastir, Monastir 5000, Tunisia

**Keywords:** *S. aureus*, whole genome sequencing, multidrug-resistant, antibiotic resistance genes

## Abstract

*Staphylococcus* species are major pathogens with increasing importance due to the rise in antibiotic resistance. Whole genome sequencing and genome-scale annotation are promising approaches to study the pathogenicity and dissemination of virulence factors in nosocomial methicillin-resistant and multidrug-resistant bacteria in intensive care units. Draft genome sequences of eight clinical *S. aureus* strains were assembled and annotated for the prediction of antimicrobial resistance genes, virulence factors, and phylogenetic analysis. Most of the studied *S. aureus* strains displayed multi-resistance toward the tested drugs, reaching more than seven drugs up to 12 in isolate S22. The *mecA* gene was detected in three isolates (S14, S21, and S23), *mecC* was identified in S8 and S9, and *blaZ* was commonly identified in all isolates except strain S23. Additionally, two complete mobile genomic islands coding for methicillin resistance SCCmec Iva (2B) were identified in strains S21 and S23. Numerous antimicrobial resistance genes (*nor*A, *norC*, *MgrA*, *tet*(45), APH(3′)-IIIa, and AAC(6′)-APH(2″)) were identified in chromosomes of different strains. Plasmid analysis revealed the presence of *blaZ*, *tetK*, and *ermC* in different plasmid types, located in gene cassettes containing plasmid replicons (rep) and insertion sequences (IS). Additionally, the aminoglycoside-resistant determinants were identified in S1 (APH(3′)-IIIa), while AAC(6)-APH(2″) was detected in strains S8 and S14. The trimethoprim (*dfrC*) resistance gene was detected in *S. aureus* S21, and the fosfomycin (*fosB*) resistance gene was detected only in *S. aureus* S14. We also noted that *S. aureus* S1 belongs to ST1-t127, which has been reported as one of the most frequent human pathogen types. Additionally, we noted the presence of rare plasmid-mediated mecC-MRSA in some of our isolates.

## 1. Introduction

*Staphylococcus aureus* is a significant human pathogen that causes severe hospital infections. Methicillin-resistant *S. aureus* (MRSA) is one of the most important causal organisms of nosocomial infections, posing a significant risk to persons with weakened immune systems [1]. Alghaithy et al. [2] reported the prevalence of nasal carriage of *S. aureus* to be about 26.1% in the community and 25.4% in healthy hospital and non-hospital staff in hospitals in Abha (Saudi Arabia). Furthermore, during a 5-year period, from January 2015 to December 2019, Bazaid and colleagues [3] reported a 12% prevalence of *S. aureus* related to urinary tract infections in patients from two main hospitals in Ha’il, Saudi Arabia. In addition, *S. aureus* was reported to cause several infections such as osteoarticular infections, endocarditis, deep soft-tissue infections, and food poisoning [4,5,6,7]. Multidrug-resistant staphylococci may persist in the hospital environment and counteract drugs and biocides by generating a biofilm or by conversion to an atypical form [8]. The pathogenicity of *Staphylococcus* isolates is associated with the production of various virulence factors expressed by chromosomal genes or mobile genetic elements [9], as well as the combined action of different components of the bacterial surface. These factors code for toxins, enzymes, and cell adhesion and invasion factors, thus allowing the bacterium to fight the immune system, adhere to cells, disseminate in the body, form protective biofilms, develop resistance to various antibiotics, and use the available nutrients and energy [10]. Due to the genetic flexibility of *S. aureus*, various drug-resistant strains have emerged, causing a serious therapeutic problem [11]. In fact, *S. aureus* has developed resistance to methicillin because it harbors a genetic element, staphylococcal cassette chromosome mec (SCCmec), which carries the methicillin resistance gene (*mec*A) responsible for methicillin and penicillin resistance [12]. According to Moussa and Shibl [13], the *mec*A gene was detected in all strains phenotypically resistant to methicillin recovered from outpatient clinics in Riyadh, Saudi Arabia. Fusidic acid is a steroid antibiotic that has been used to treat *S. aureus* since the 1960s. Unfortunately, high fusidic acid use may result in the rapid development of resistance [14]. Fusidic acid resistance is frequent in Middle Eastern/Arabic Gulf countries and is mainly caused by plasmid-borne fusB/far1 [15,16] or SCC-associated fusC [17]. According to Albarrag et al. [18], 47% of analyzed MRSA isolated from a nursing home in Riyadh developed multiple-drug resistance (MDR) and were *mecA*-positive, and the SCCmec types were as follows: SCCmec IVc (41.18%), SCCmec V (29.41%), and SCCmec IVa (11.76%).

The pathogenicity of *Staphylococcus* spp. is amplified by its ability to form a biofilm that proliferates on various inert or biological surfaces. In fact, the formation of a biofilm reinforces adhesion to materials and protects the bacteria from immune defenses and the action of antimicrobial agents [19]. Several studies have shown that staphylococcal biofilm can also settle on biotic surfaces and even abiotic surfaces. Several genes implicated in adhesion and biofilm formation have been reported in *S. aureus* [20,21,22,23].

The comparative genomics technique was used to determine the evolutionary processes of clinically relevant *S. aureus* genomes and to identify areas influencing the acquisition of drug resistance and virulence factors [11]. Several staphylococci clones have been discovered internationally and regionally, and their epidemiological, clinical, and genetic characteristics have been evaluated [24]. Whole genome sequencing (WGS) enables the comparison of entire genomic DNA sequences and detects genetic variation across species [25], and it is considered a high-resolution approach for confirming outbreaks, studying pathogenesis, predicting resistance, assessing virulence, and typing microbial species [26]. This approach has been used to investigate the epidemiology of MRSA and its spread within hospitals as well as from hospitals to the general population [27].

Hence, the main aims of this study were to investigate whole genome sequencing, typing, and prediction of resistome and virulome in some clinical multidrug-resistant *S. aureus* strains associated with human infections.

## 2. Materials and Methods

### 2.1. Isolation of Multidrug-Resistant S. aureus

Various clinical multidrug-resistant *S. aureus* strains (Table 1) were collected from the microbiology laboratory at King Khalid hospital, Ha’il, Saudi Arabia, in March 2021. Samples from wounds, throat, sputum, and pleural fluid were plated in blood and MacConkey agar plates (Oxoid, Basingstoke, UK) and then incubated at 37 °C for 24 h. The purity of the suspected *S. aureus* isolates was then confirmed by sub-culturing on mannitol salt agar. Patient information including gender and location were collected from hospital records. The study was approved by the Ethics Committee at Ha’il Affairs (reference H-08-L-074). A consent form was not required because isolates were collected from the laboratory with no interaction with patients. Patient privacy and confidentiality of data were maintained in accordance with The Declaration of Helsinki.

### 2.2. Antimicrobial Susceptibility Testing

Antimicrobial susceptibility of the suspected *S. aureus* strains was achieved using a BD Phoenix™ M50 instrument (Becton, Dickinson and Co., Franklin Lakes, NJ, USA) as previously reported [28]. Susceptibility of the identified *S. aureus* was tested toward several antibiotics including ampicillin, amoxicillin–clavulanate, cefuroxime, cefoxitin, clindamycin, ciprofloxacin, daptomycin, erythromycin, fusidic acid, gentamicin, imipenem, mupirocin, linezolid, penicillin G, trimethoprim-sulfamethoxazole, nitrofurantoin, tetracycline, teicoplanin, vancomycin, rifampicin, moxifloxacin, and oxacillin. The results of the drug-susceptibility tests were interpreted according to Clinical and Laboratory Standards Institute (CLSI) guidelines document M100S-26 [29]. Two indices were used to interpret the obtained results, including the multiple antibiotic resistance index and the antibiotic resistance index as previously described by Abdulhakeem and colleagues [30].

### 2.3. Molecular Typing of Multi-Drug-Resistant S. aureus

#### 2.3.1. *S. aureus* DNA Extraction

Total DNA was extracted from overnight 24 h bacterial culture growth. The guanidine chloride procedure was used for DNA extraction as detailed previously [31]. DNA quality and quantity were determined by gel-electrophoresis and Qubit (Thermo Scientific, Waltham, MA, USA).

#### 2.3.2. Whole Genome Sequencing, Typing, and Prediction of Resistome and Virulome

The sequencing of the genomes of the selected strains was done by Novogene Company (Hong Kong, China) using Illumina HiSeq 2500 (Illumina, San Diego, CA, USA), and paired-end 150 bp reads with 100X coverage were achieved. Genome sequences were reassembled using SPAdes version 3.7 software [31,32]. Plasmids were assembled using plasmidSPAdes tool v3.15.4, applying different k-mer sizes (21, 33, and 55) [33]. The assembled contigs were submitted to the MLST 2.0 and PubMLST database [34] for species identification. The assembled genomes were run through the annotation pipeline PATRIC server and the NCBI Prokaryotic Genome Annotation Pipeline (PGAP) [35]. The determination of isolate sequence types (ST) and *S. aureus*-specific staphylococcal protein A (spa) genes were achieved using the global platform for genomic surveillance (Pathogenwatch) and spaTyper 1.0, respectively [36]. MLST clonal complexes (CCs) were determined according to the Ridom StaphType database’s previous publications [37,38]. Plasmids generated from plasmidSPAdes were identified using BLASTn and Mobile Element Finder [39]. Genomic and plasmid-mediated antimicrobial resistance were identified using Resistance Gene Identifier (RGI) and ResFinder [40]. Point mutations associated with drug resistance were screened using the RGI database. Staphylococcal chromosomal cassettes *mec* (SCCmec) were identified using SCCmec Finder 1.2. Virulence genes were identified from the assembled contigs using the Virulent Factor Database (VFDB, Version: 2016.03) applying MRSA252 as a reference genome. Phages were screened by PHASTER (PHAge Search Tool Enhanced Release) [41].

#### 2.3.3. Pan-Genome Analysis and Phylogenomic Tree

Pan-genome analysis of all isolates’ genomes was conducted using Roary V. 3.11.2 software, and the general feature format (GFF) files generated from Prokka [42] annotation were used as input for Roary with default settings [43]. The alignment FASTA file generated from Roary was used as input for RaXML [44] for building the phylogenetic tree, which was then visualized by the interactive visualizer of genome phylogenies (Phandango) [45]. The Gene presence–absence file generated from Roary was uploaded to Scoary [46] to calculate the association between gene presence and absence with different traits such as methicillin resistance and source of samples.

## 3. Results and Discussion

### 3.1. Antimicrobial Susceptibility of the Isolated S. aureus

In this study, eight clinical *S. aureus* strains were tested for their antibiotic resistance profile using a BD Phoenix™ M50 instrument. Based on the minimum inhibitory concentration (MIC) values obtained, our results showed that almost all tested *S. aureus* strains were resistant to 3 to 12 antibiotics used (out of 21 tested). Interestingly, all tested *S. aureus* strains were highly resistant to cefoxitin (87.5%), Penicillin G (87.5%), and erythromycin (62.5%). In addition, the tested strains were completely resistant to ampicillin and cefotaxime, and sensitive to tigecycline, nitrofurantoin, and rifampicin. All these data are summarized in Table 2 in below.

Using the multiple antibiotic resistance index (MARI) and the antibiotic resistance index (ARI), results obtained showed that the ARI ranged from 0 to 1, while the MARI varied from 0.190 for *S. aureus* (S14) isolated from eye infection to 0.571 for *S. aureus* (S22) isolated from pleural fluid (Table 3).

### 3.2. Genome Composition and Genomic Variation

Genomes were assembled with an average of 2.75 Mb (2.74–2.85 Mb), at least 2601 predicted coding sequences (CDS), and a GC content of 32.7% for all isolates (Table 4); these results are consistent with *S. aureus* genome characteristics [47]. Isolates belonged to ST1 (S1 and S20), ST97 (S8 and S9), ST121 (S14), ST22 (S21), ST291 (S22), and ST6 (S23). Isolate S1 belonged to ST1-t127, which has been reported as one of the most frequent human pathogen types [48].

Data obtained from Saudi Arabia showed that ST239 is the most common in Riyadh and Dammam, located in the eastern region of Saudi Arabia [49], while in the western region this clone is not common [50], which is consistent with our findings. All clones identified here have been identified sporadically in the western region by Al-Zahrani et al. [51].

### 3.3. Prediction of Antimicrobial Resistance Mechanism in the Studied S. aureus

The isolates were identified with resistance genes to several antimicrobial classes as shown in Table 2 and Table 3; these genes are associated with phenotypic findings in which the isolates were multidrug-resistant to different classes of antibiotics as shown in Table 1. Genes responsible for beta-lactam resistance (*bla*Z, *mec*A, and *mec*C) were identified in the studied isolates: the *mec*A gene was detected in three MRSA isolates (S14, S21, and S23), *mec*C was identified in S8 and S9 isolates, and *bla*Z was commonly identified in all isolates except S23 (Table 5). Interestingly, we identified the presence of *mec*C-MRSA for the first time in our region; *mec*C is a new divergent from *mec*A usually associated with animal transmission, and these patients are usually from rural areas [51].

Eight antimicrobial-resistance-associated genes were identified in all of the isolates: the quinolone resistance genes (*nor*A and *nor*C) and their regulators (*Mgr*A for *nor*C and *arl*S for *nor*A), the major facilitator superfamily MDR efflux pump in *S. aureus* (*Lmr*S), the multidrug efflux pump (*sdr*M), the *mep*A gene which acts as a repressor for multidrug export protein, and the *Sep*A gene that confers resistance to disinfecting agents and dyes [52]. *Sep*A was also demonstrated to have induction activity on biofilm accumulation [53].

Although isolate S1 lacked the *mec*A and *mec*C genes, it exclusively harbored genes associated with resistance to different drug classes, including the tetracycline resistance gene (*tet*(45)), aminoglycoside resistance gene (APH(3′)-IIIa), nucleoside antibiotic (SAT-4), and erythromycin resistance genes (*ermC*) (Table 3). A total of 4 out of 8 (50%) isolates were positive for a gene (*fusC*) conferring resistance to fusidane antibiotic; a high prevalence of *fusC* in *S. aureus* has been documented recently in Saudi Arabia [54]. Aminoglycosides are among the important antibiotics that are used to treat a range of bacterial infections, especially those caused by *Staphylococcus* species [54]. Here, the aminoglycoside-resistant determinants were identified in S1 (APH(3′)-IIIa), and the AAC(6′)-APH(2″) was detected in strains S8 and S14. Additionally, strains S8 and S14 are MRSA and harbor multiple genes associated with resistance to different classes of antibiotics (Table 5). The presence of additional aminoglycoside-resistance genes will limit the therapeutic options, and this makes treatment for such isolates more challenging and eventually leads to the continuing spread of MRSA strains.

Point mutations (L27F, A100V, E291D, and T396N) associated with phosphonic acid resistance have been identified in isolate S14, while isolate S23 was identified with a fluoroquinolone-resistant *parC* gene mutation (S80F), GlpT gene mutations (F3I, A100V) conferring resistance to fosfomycin, and a *gyrA* gene mutation (S84L) conferring resistance to fluoroquinolones (Supplementary Table S1).

### 3.4. Plasmid-Mediated Antimicrobial Resistance Genes

Plasmid analysis revealed the presence of *bla*Z, tetK, and *ermC* in different plasmid types, located in gene cassettes containing plasmid replicons (rep) and insertion sequences (IS) (Table 6). The presence of these genes in a plasmid in association with plasmid replicons and insertion sequences will facilitate their distribution and transmission between resistant and susceptible isolates, which is reflected in their high distribution in our isolates. A gene cassette consisting of rep20, *blaZ*, and ISSau6 was identified in plasmid *p*USA300HOUMS circulating in both *S. aureus* S8 and S9 (Table 6). The *blaZ* gene is a plasmid-mediated β-lactamase causing penicillin resistance and could also resist all β-lactam antibiotics [55,56]. The co-existence of the *blaZ* gene with the chromosomally mediated *mec* genes could be the reason for the high resistance of β-lactam antibiotics observed in our isolates. As presented in Table 4, we found isolate S22 with two rep genes (rep5 and rep16) and *blaZ* in one plasmid (*p*LDNT_611); the co-occurrence of two rep genes in one plasmid could indicate the occurrence of recombination between different *S. aureus* plasmids [57]. A gene encoding macrolides–lincosamides–streptogramin resistance (*ermC*) and rep10 were identified in plasmid pE5 circulating in three isolates: S1, S14, and S23. These results indicate that these isolates have acquired these genes from others via plasmids, which is in accordance with previous studies [58,59]. Horizontal transfer of the *ermC* gene in a small plasmid was commonly documented in animal and human staphylococcal species [59]. In accordance with a previous study conducted in Saudi Arabia, the prevalence of *ermC* in *S. aureus* isolates was 28.8% in this study [60]. The MRSA-S14 strain harbored an additional plasmid (pUSA02) that carried *tetK*; the presence of *tetK* in *S. aureus p*USA02 plasmid has been documented in a previous study [61].

### 3.5. Analysis of Staphylococcal Chromosomal Cassettes mec (SCCmec)

**Figure 1:**
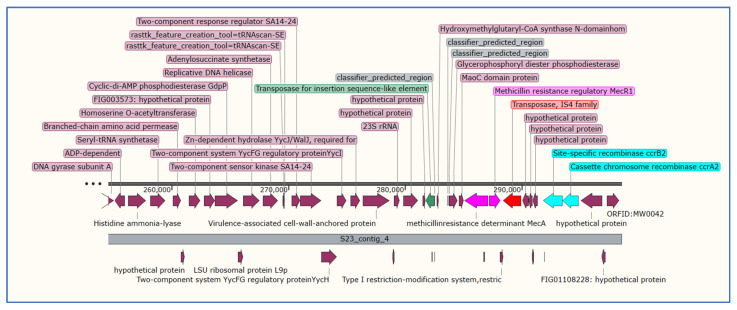
Staphylococcal chromosomal cassettes *mec* (SCCmec) (IVa(2B)) identified in isolate S21. The cassette chromosomal recombinase genes are in turquoise, the *mec*A gene and its regulator are in pink, transposase is in red, Transposase in green, and recombinase in cyan, purple 9ndicate the rest of genes.

The SCCmec is a mobile genomic island coding for methicillin resistance, classified into different subtypes according to the SCCmec gene cassette arrangement [62]. In this study, two complete SCCmec IVa(2B) were identified in isolates S21 and S23, while isolates S1, S8, S9, and S14 were documented with incomplete cassettes (Table 7, Figure 1). Isolate S21 belongs to ST22 and harbors the toxic shock syndrome gene (TSST-1), and CC22-MRSA-IV is a pandemic MRSA strain mainly found in Western Europe; recently, CC22-MRSA-IV with or without the TSST gene has been commonly identified in different Middle Eastern countries including Saudi Arabia [60].

Additionally, CC22-MRSA-IV strains positive for TSST are epidemically reported in Gaza, Palestine [63], which suggests an epidemiological link to Saudi Arabia. Isolate S23 belonged to ST6 and has spa type 304 (CC6-ST6-IV/t304), which is called the Western Australia (WA) MRSA-51 clone; this clone is reported dominantly in different Gulf countries, including Saudi Arabia, UAE, Kuwait, and Oman, with a prevalence of more than 36% in Oman and UAE [64]. The presence of incomplete SCCmec cassettes suggests a probable sequence deletion or insertion due to an unknown process [65].

### 3.6. Pan-Genome Analysis

**Figure 2:**
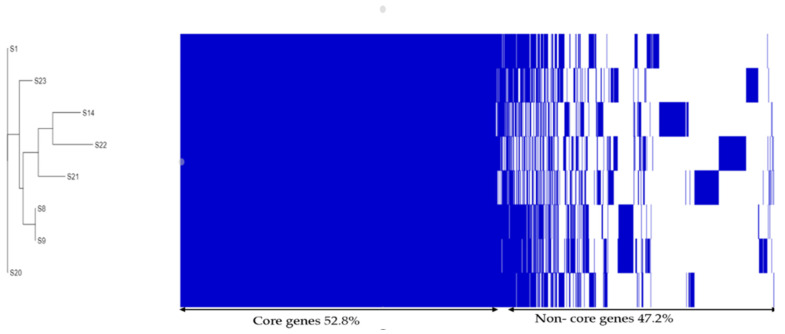
Core-genome phylogenetic analysis showing core and non-core genes in *S. aureus* isolates. On the left, the phylogenetic tree is built from 3837 core genes of the isolates. The blue heat map shows core genes (dark blue) and the blue boxes and white gaps show non-core genes. Each row represents the aligned sample.

Analysis of the staphylococcal pan-, core-, and accessory genomes revealed the presence of a total of 3837 genes, from which 2027 genes represent the core genes that are shared in >99% of all isolates, indicating their high similarity, ribosomes, and proteins associated with biogenesis [66,67]. A total of 992 genes were identified as shell genes present in >15% of the isolates; cloud genes represented 0–15% (818 genes), and soft-core genes were not identified in our studied isolates (Table 8). More details about core genes are represented in Supplementary File Table S2 and Figure 2.

### 3.7. Molecular Analysis of Virulence Factors in the Studied S. aureus

#### 3.7.1. Detection of Panton–Valentine Leukocidin Gene

Panton–Valentine leukocidin (PVL) toxin is a virulence factor in *S. aureus* associated with deep skin and soft-tissue infections (furunculosis, cutaneous abscesses, and severe necrotizing pneumonia) [68,69,70]. In 1932, Panton and Valentine defined the PVL as a virulence factor in the family of synergohymenotropic toxins in *S. aureus* [71]. PVL is a toxin with two components that are encoded by the prophage-associated genes *luk*F-PV and *luk*S-PV [72].

We detected two PVL (lukF-PV and lukS-PV) in the *S. aureus* isolate S14 on the orf02119 and orf02120, respectively (Supplementary File Tables S1 and S2).

Recently, El-Deeb et al. [73] analyzed the whole genome of nine methicillin-resistant staphylococci (MRS) collected in Eastern Province, Saudi Arabia, and they found that only isolate SA1 recovered from goat’s milk had the PVL toxin gene and the *Staphylococcus* enterotoxin B gene *seb*. Ullah et al. [70] conducted a genomic investigation of an *S. aureus* strain isolated from Pakistan and discovered that the strain has many prophage-associated virulence factors, including PVL and toxic shock syndrome toxin (TSST). Additionally, all 52 isolates of *S. aureus* isolated from the vascular accesses in hemodialysis patients at Assiout University Hospitals were negative for the PVL gene [74]. Moreover, Alghizzi and Shami [75] analyzed 112 *S. aureus* strains isolated from raw milk and cheese and were unable to detect the PVL gene in any of the analyzed strains [75].

#### 3.7.2. Detection of Genes Related to Iron Uptake System in *S. aureus*

**Figure 3:**
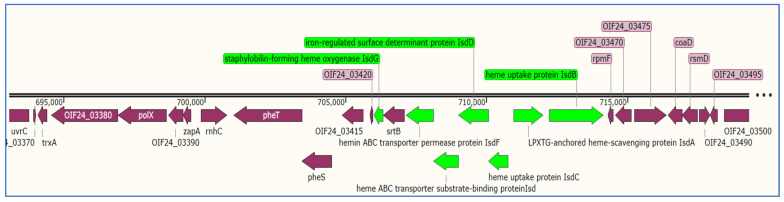
Map of *isd* locus and surrounding chromosomal genes (purple) in isolate S1; *isd* genes are colored in green.

*S. aureus* needs iron to survive, multiply, and infect cells, hence it has evolved specific proteins to steal heme from its host. The iron surface determinant (Isd) system is a protein family that obtains nutritional iron from the host body, allowing the bacteria to multiply during infection [76]. Staphylococcal cell-surface proteins (IsdA, IsdB, and IsdH) are assumed to channel their molecular payload to IsdC, which subsequently facilitates the transfer of the iron-containing nutrition to the membrane translocation system IsdDEF [77]. According to Valenciano-Bellido et al. [76], IsdH, a surface protein, binds to hemoglobin (Hb) and absorbs the heme moiety carrying the iron atom. A cluster of iron surface determinants (Isd) have been identified similarly in all isolates, which includes LPXTG-anchored heme-scavenging protein (IsdA), heme uptake protein (IsdB), heme uptake protein (IsdC), iron-regulated surface determinant protein (IsdD), heme ABC transporter substrate-binding protein (IsdE), and hemin ABC transporter permease protein (IsdF) (Figure 3). The staphylobilin-forming heme oxygenase (IsdI) was detected separately in another contig. The Isd locus, which is required for the acquisition of iron from hemoglobin, was identified in this study in all of the isolates; the common presence of this locus in different staphylococcus species has been documented previously [2].

#### 3.7.3. Detection of Autolysin Genes in the Studied *S. aureus* Strains

Autolysin (Atl) is considered a peptidoglycan hydrolase (PGH) that is primarily responsible for the breakdown of the bacterial cell wall as well as daughter cell separation during cell division [78,79]. Atl of *S. aureus* is a cell-surface-associated peptidoglycan hydrolase containing amidase and glucosaminidase domains. Atl enzymes have been linked to biofilm development as well as staphylococcal adhesion to host extracellular and plasma proteins [80]. More recently, Zheng and colleagues [81] reported that Atl controls the sorting of LukAB from the cell envelope to the extracellular milieu. In our study, autolysin was detected in seven clinical *S. aureus* strains as well as the control strain (Supplementary Tables S1 and S2).

#### 3.7.4. Detection of Enterotoxin and Exotoxin in the Studied *S. aureus*

Supplementary Table S1 lists genes related to staphylococcal enterotoxin A (*sea*) (37.5%), B (*seb*) (25%), G (*seg*) (25%), H (*seh*) (25%), I (*sei*) (25%), and enterotoxin-like K (*selk*) (25%), M (*selm*) (25%), N (*seln*) (25%), O (*selo*) (25%), Q (*selq*) (25%), and U (*selu*) (25%), while the following genes were absent; C (*sec*), D (*sed*), E (*see*), J (*sej*), L (*sell*), P (*selp*), and R (*selr*). According to Alghizzi and Shami [75], *seh* enterotoxin gene (51%) showed the highest ratio among the analyzed ones followed by *see* (27.5%), *sem* (21.6%), *seo* (19.6%), *sea* and *sen* (17.6%), *seb* (13.7%), *seg* (11.8%), *sed* and *sei* (3.9%), and lastly *sec* and *sek* (1.96%).

We noted the absence of *sec*, se*lk*, *see*, *sej*, *sell*, *selp*, and *selr* (Supplementary Table S1). Similarly, neither *sej* nor *sel* existed in any of the MRSA isolates [75]. I have been demonstrated the most prevalent enterotoxin gene from *S. aureus* strains isolated from cell phones were *sea* (30%) followed by the *sec* gene (2.5%), while *sed* and *seb* genes not detected in any of the isolates [82]. Moreover, the analysis of 88 MRSA collected from 5 hospitals in Makkah demonstrated the presence of 2.3% *S. aureus* positive for the *etb* toxin gene, and none of the tested strains harbored the *eta* toxin gene [83]. According to Hamdan-Partida et al. [84], the *seb* gene (63.8%), which was more prevalent in strains isolated from the pharynx, and the *tsst* gene (57.8%), which was more prevalent in nose strains, were the most common toxin genes discovered. Additionally, we did not detect *yent1* (Enterotoxin *Yent1*) and *yent2* (Enterotoxin *Yent2*) genes. Moreover, the exfoliative toxin type A (*eta*), B (*etb*), C (*etc*), and D (*etd*) were also not detected in the analyzed samples (Supplementary Table S1).

#### 3.7.5. Detection of Toxic Shock Syndrome Toxin in *S. aureus*

**Figure 4:**
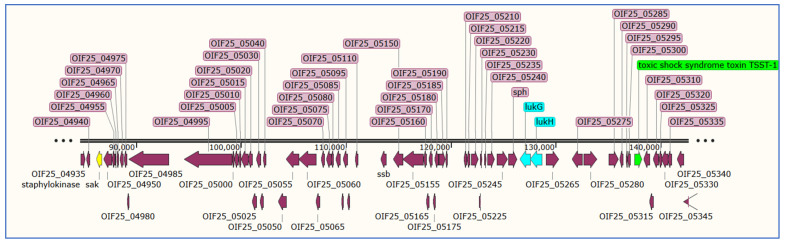
Map of a group of virulence genes located at the same contig in isolate S21 and surrounded by other staphylococcal proteins (purple). The green color shows the toxic shock syndrome toxin (*TSST*-1); leukocidins (*lukGH*) are colored in turquoise, the staphylokinase (*sak*) gene is colored in yellow, the leukocidins G and H are in cyan color, the rest of genes are colored in purple.

The toxic shock syndrome toxin 1 was considered among the main virulence factors elaborated by *S. aureus* associated with scalded skin syndrome [85]. *Tsst*-1 is frequently seen in conjunction with septic shock and toxic shock syndromes, and exfoliative toxins are common in isolates producing staphylococcal scalded skin syndrome [86]. In our investigation, the toxic shock syndrome toxin (*tsst*-1) was detected only in one *S. aureus* isolate (S21) located in the orf01006 (Supplementary Tables S1 and S2, Figure 4). In 2016, Ahmed [83] used multiplex PCR to analyze 88 MRSA isolates collected from 5 hospitals in Makkah, and they reported the presence of 3.4% positive strains for toxic shock syndrome toxin. Additionally, Udo et al. [87] demonstrated the presence of *tsst* in 23 out of 37 MRSA collected from patients in Kuwait hospitals.

## 4. Conclusions

In this study, eight clinical multidrug-resistant *S. aureus* strains were collected from the microbiology laboratory at King Khalid hospital, Ha’il, Saudi Arabia. The analysis of whole genomes revealed the presence of virulent and multidrug resistance determinants in the studied *S. aureus* strains. Interestingly, according to our literature survey, no previous study has been conducted in Saudi Arabia that has documented the presence of plasmid-mediated mecC-MRSA, so this report is considered the first one in our region. Additionally, *mecA*, *norA*, and *norC*, *MgrA, tet(45)*, *APH(3′)-IIIa*, *blaZ*, *tetK*, and *AAC(6′)-APH(2″)* were also identified in the isolates. The pathogenic TSST-1-positive Western European MRSA strain (CC22-MRSA-IV) was also reported in this study.

## Figures and Tables

**Table 1:** Type and antibiotic resistance profile of *S. aureus* collected in this study with patient gender and location.

Strain	Gender	Location	Sample
**S1**	Male	Ward	Wound
**S8**	Female	Ward	Wound
**S9**	Male	ICU	Throat swab
**S14**	Male	PICU	Eye swab
**S20**	Male	ICU	Sputum
**S21**	Female	Ward	Wound
**S22**	Male	Ward	Pleural Fluid
**S23**	Male	Ward	Wound

**Table 2:** Antibiotic susceptibility profiles obtained by using a BD Phoenix™ M50 instrument.

Strain	Resistance Profile
**S1**	Cefoxitin; Cefotaxime; Ampicillin; Penicillin G; Oxacillin; Trimethoprim; Erythromycin; Ciprofloxacin; Tetracycline
**S8**	Gentamicin; Cefoxitin; Cefotaxime; Ampicillin; Penicillin G
**S9**	Gentamicin; Cefoxitin; Cefotaxime; Ampicillin; Penicillin G; Oxacillin
**S14**	Cefotaxime; Ceftaroline; Ampicillin
**S20**	Cefoxitin; Cefotaxime; Ceftaroline; Ampicillin; Penicillin G; Teicoplanin; Vancomycin; Clindamycin; Erythromycin; Linezolid; Tetracycline
**S21**	Cefoxitin; Cefotaxime; Ceftaroline; Ampicillin; Penicillin G; Oxacillin; Daptomycin; Trimethoprim; Vancomycin
**S22**	Cefoxitin; Cefotaxime; Ampicillin; Penicillin G; Teicoplanin; Vancomycin; Clindamycin; Erythromycin; Ciprofloxacin; Levofloxacin; Moxifloxacin; Tetracycline
**S23**	Cefoxitin; Cefotaxime; Ampicillin; Penicillin G; Oxacillin; Clindamycin; Erythromycin

**Table 3:** Multiple antibiotic resistance index (MARI) and antibiotic resistance index (ARI) distribution in the six *S. aureus* strains tested.

Antibiotics Tested	S1	S8	S9	S14	S20	S21	S22	S23	ARI
**Gentamicin**	S	R	R	S	S	S	S	S	2/8 = 0.25
**Cefoxitin**	R	R	R	S	R	R	R	R	7/8 = 0.875
**Cefotaxime**	R	R	R	R	R	R	R	R	8/8 = 1
**Ceftaroline**	S	S	S	R	R	R	I	S	3/8 = 0.375
**Ampicillin**	R	R	R	R	R	R	R	R	8/8 = 1
**Penicillin G**	R	R	R	S	R	R	R	R	7/8 = 0.875
**Oxacillin**	R	S	R	S	S	R	S	R	4/8 = 0.5
**Daptomycin**	S	S	S	S	S	R	S	S	1/8 = 0.125
**Trimethoprim**	R	S	S	S	S	R	S	S	2/8 = 0.25
**Teicoplanin**	S	S	S	S	R	S	R	S	2/8 = 0.25
**Vancomycin**	S	S	S	S	R	R	R	S	3/8 = 0.375
**Clindamycin**	S	S	S	S	R	S	R	R	3/8 = 0.375
**Erythromycin**	R	S	S	R	R	S	R	R	5/8 = 0.625
**Linezolid**	S	S	S	S	R	S	S	S	1/8 = 0.125
**Nitrofurantoin**	S	S	S	S	S	S	S	S	0/8 = 0
**Ciprofloxacin**	R	S	S	S	S	S	R	S	2/8 = 0.25
**Levofloxacin**	S	S	S	S	S	S	R	S	1/8 = 0.125
**Moxifloxacin**	S	S	S	S	S	S	R	S	1/8 = 0.125
**Rifampin**	S	S	S	S	S	S	S	S	0/8 = 0
**Tetracycline**	R	S	S	S	R	S	R	S	3/8 = 0.375
**Tigecycline**	S	S	S	S	S	S	S	S	0/8 = 0
**MARI**	0.428	0.238	0.285	0.190	0.523	0.428	0.571	0.333	

**Table 4:** Genome characteristics of the studied *S. aureus* and assembly statistics.

Isolate	ST	Spa Type	MLST CCs	Genome Length	No of Contigs	Coverage	N50	GC	CDS	tRNA	rRNA
**S1**	1	t127	CC01	2,806,634	21	438	336,511	32.7%	2659	54	4
**S8**	97	t189	CC97	2,758,541	32	463	300,844	32.7%	2632	56	3
**S9**	97	t2297	CC97	2,853,553	48	403	286,668	32.7%	2777	55	3
**S14**	121	t314	CC121	2,778,595	61	331	113,582	32.7%	2634	58	4
**S20**	1	t5388	CC01	2,785,405	14	331	547,174	32.7%	2645	55	3
**S21**	22	t845	C22	2,742,174	44	471	173,813	32.7%	2607	53	5
**S22**	291	t3649	CC291	2,755,471	34	526	211,298	32.7%	2632	53	3
**S23**	6	t304	CC6	2,750,772	26	485	298,524	32.7%	2601	57	4
Biosample/Accession number: S1 (SAMN31278742/JAOXRK000000000); S8 (SAMN31278743/JAOXRJ000000000); S9 (SAMN31278744/JAOXRI000000000); S14 (SAMN31278745/JAOXRH000000000); S20 (SAMN31278746/JAOXRG000000000); S21 (SAMN31278747/JAOXRF000000000); S22 (SAMN31278748/JAOXRE000000000); S23 (SAMN31278749/JAOXRD000000000).											

**Table 5:** Predicted antimicrobial resistance genes among the studied *S. aureus*.

Gene	Resistance Mechanism	S1	S8	S9	S14	S20	S21	S22	S23
*mec*A	Antibiotic target replacement				x		x		x
*mec*C	Antibiotic target replacement		x	x					
*arl*R	Antibiotic efflux	x	x	x	x	x	x	x	x
*arl*S	Antibiotic efflux	x				x	x		x
*S. aureus nor*A	Antibiotic efflux	x	x	x	x	x	x	x	x
*mgr*A	Antibiotic efflux	x	x	x	x	x	x	x	x
dfrC	Antibiotic target replacement						x		
fusC	Antibiotic target protection	x	x	x	x				
*S. aureus* FosB	Antibiotic inactivation				x				
*mep*R	Antibiotic efflux	x	x	x	x	x	x	x	x
*APH*(3′)-IIIa	Antibiotic inactivation	x							
*AAC(*6′*)-APH(*2*″)*	Antibiotic inactivation		x		x				
*nor*C	Antibiotic efflux	x	x	x	x	x	x	x	x
*S. aureus* LmrS	Antibiotic efflux	x	x	x	x	x	x	x	x
*sep*A	Antibiotic efflux	x	x	x	x	x	x	x	x
*sdr*M	Antibiotic efflux	x	x	x	x	x	x	x	x
*tet*(45)	Antibiotic efflux	x							
*tet*K	Antibiotic efflux				x				
PC1 beta-lactamase (*bla*Z)	Antibiotic inactivation	x	x	x	x	x	x	x	
*SAT*-4	Antibiotic inactivation	x							
*Erm*C	Antibiotic target alteration	x							x

**Table 6:** Plasmids identified in the studied *S. aureus* associated with drug resistance genes and mobile elements.

Strain	Plasmid	Contig	Coverage	Plasmid Identity	Reference	Genes		
						Name	Position in Contig	Identity
**S1**	*p*E5	1	1573	99%	M17990.1	*erm(C)*	1542–2276	100%
						*rep10*	251–727	100%
**S8**	*p*USA300HOUMS	1	697	99%	CP000732.1	*rep20*	19,264–20,253	100%
						*blaZ*	10,806–9961	100%
						*ISSau6*	10,806–9961	100%
**S9**	*p*USA300HOUMS	3	426	99%	CP000732.1	*rep20*	2896–3885	100%
						*blaZ*	13,996–14,841	100%
						*ISSau6*	13,996–14,841	100%
**S14**	*p*USA02	2	1477	99%	CP000257.1	*tetK*	1425–46	100%
						*rep7a*	3379–4323	100%
	*p*E5	3	5.73	99%	M17990.1	*rep10*	512–988	100%
						*erm(C)*	1803–2537	100%
**S20**	*p*ER07993.3A.1	1	648	99%	CP049391.1	*rep5a*	1669–2529	100%
						*blaZ*	8989–9834	100%
**S21**	-	-	-	-	-	*-*	-	-
**S22**	*p*LDNT_611	1	897	99%	CP080252.1	*rep5a*	1066–206	100%
						*rep16*	2357–3100	100%
						*blaZ*	13,545–14,390	100%
**S23**	*p*E5	2	2296	99%	M17990.1	*rep10*	1746–2222	100%
						*erm(C)*	197–931	100%

**Table 7:** Staphylococcal chromosomal cassettes *mec* (SCCmec) identified in the studied *S. aureus*.

Isolate	SCCmec Type	SCCmec Gene Cassettes	Identity (%)	Ref. Coverage	Contig No	Contig Position
**S1**	N/A	ccrB1:1:COL:CP000046	92.25	1626/1625	S1_contig_2	63,208–64,832
		ccrA1:1:COL:CP000046	94.37	1350/1350	S1_contig_2	64,854–66,203
**S8**	N/A	ccrC1-allele-8:1:AB462393	99.94	1677/1677	S8_contig_3	1522–3198
		mec-class-C2:3:AB478780	99.91	2290/2398	S8_contig_18	2493–4782
**S9**	N/A	ccrC1-allele-8:1:AB462393	98.45	1677/1677	S9_contig_20	1462–3138
		mec-class-C2:3:AB478780	100	2198/2398	S9_contig_23	2400–4597
		ccrC1-allele-2:1:AB512767	100	1680/1680	S9_contig_1	7153–8832
**S14**	N/A	mec-class-C2:5:AB505629	99.86	4408/4408	S14_contig_44	295–4702
		ccrC1-allele-8:1:AB462393	99.94	1677/1677	S14_contig_14	55,301–56,977
**S20**	No SCCmec					
**S21**	IVa(2B)	mecA:5:CP000046	100	2007/2007	S21_contig_18	1366–3372
		IS1272:2:AB033763	99.68	1550/1585	S21_contig_34	1–1550
		subtype-IVa(2B):1:CA05:AB063172	100	1491/1491	S21_contig_20	27,585–29,075
		dmecR1:1:AB033763	100	987/987	S21_contig_18	280–1266
		ccrA2:7:81108:AB096217	100	1350/1350	S21_contig_20	33,027–34,376
		ccrB2:9:JCSC4469:AB097677	99.94	1650/1650	S21_contig_20	34,377–36,026
**S22**	No SCCmec					
**S23**	IVa(2B)	subtype-a(2B):1:CA05:AB063172	99.93	1491/1491	S23_contig_16	28,081–29,571
		mecA:12:AB505628	100	2010/2010	S23_contig_4	285,112–287,121
		dmecR1:1:AB033763	100	987/987	S23_contig_4	287,218–288,204
		IS1272:3:AM292304	100	1843/1843	S23_contig_4	288,193–290,035
		ccrB2:9:JCSC4469:AB097677	99.94	1650/1650	S23_contig_4	291,877–293,526
		ccrA2:7:81108:AB096217	100	1350/1350	S23_contig_4	293,527–294,876

Abbreviations: N/A = The sequencing result was not complete and the SCCmec type was not determined.

**Table 8:** Frequency of staphylococcal isolates’ pan-genomes, categorized into core, soft-core, shell, and cloud genes.

**Core Genes**	(99% ≤ strains ≤ 100%)	2027
**Soft-core genes**	(95% ≤ strains < 99%)	0
**Shell genes**	(15% ≤ strains < 95%)	992
**Cloud genes**	(0% ≤ strains < 15%)	818
**Total genes**	(0% ≤ strains ≤ 100%)	3837

## Data Availability

The Whole Genome Shotgun project PRJNA890445 has been deposited at DDBJ/ENA/GenBank under accession numbers JAOXRK000000000, JAOXRJ000000000, JAOXRI000000000, JAOXRH000000000, JAOXRG000000000, JAOXRF000000000, JAOXRE000000000, JAOXRD000000000 for the studied strains S1, S8, S9, S14, S20, S21, S22, and S23, respectively.

## Supplementary Materials

The following supporting information can be downloaded at: https://www.mdpi.com/article/10.3390/microorganisms11051124/s1, Table S1: Details of virulence factors identified in the analyzed *S. aureus*; Table S2: Predicted virulence factors in the whole genome of the studied *S. aureus*.
