# Supplementary material for: Antibiotic Susceptibility Profiling of Human Pathogenic Staphylococcus aureus Strains Using Whole Genome Sequencing and Genome-Scale Annotation Approaches
Source: Microorganisms. 2023 Apr 26;11(5):1124. doi: 10.3390/microorganisms11051124 (PMC10221271; doi:10.3390/microorganisms11051124)
Supplement: Supplementary file 1 [file microorganisms-11-01124-s001.zip › microorganisms-2350313-supplementary.pdf]

Antibiotic susceptibility profiling of human pathogenic *Staphylococcus aureus* strains using whole genome sequencing and genome-scale annotation approaches

Mejdi Snoussi[1,2*], Emira Noumi[1,2], Nouha Bouali[1,2], Abdulrahman S. Bazaid[3], Mousa Alrashidi[1,4], Hisham N. Altayb[5,6], Kamel Chaieb[5,7]

[1] Department of Biology, College of Science, University of Hail, P.O. Box 2440, Ha'il 2440, Saudi Arabia; m.snoussi@uoh.edu.sa (M.S.); n.bouali@uoh.edu.sa (N.B.); eb.noumi@uoh.edu.sa (E.N.).

[2] Medical and Diagnostic Research Centre, University of Ha'il, Hail 55473, Saudi Arabia

[3] Department of Medical Laboratory Science, College of Applied Medical Sciences, University of Hail, Hail 55476, Saudi Arabia, ar.bazaid@uoh.edu.sa (A.S.B.)

[4] Molecular Diagnostics and Personalized Therapeutics Unit, University of Hail, Hail P.O. Box 2440, Saudi Arabia, mo.alreshidi@uoh.edu.sa (M.R.)

[5] Department of Biochemistry, Faculty of Science, King Abdulaziz University, Jeddah, Saudi Arabia; hdemmahom@kau.edu.sa (H.N.A.)

[6] Center of Artificial Intelligence in Precision Medicines, King Abdulaziz University, Saudi Arabia; kalshaib@kau.edu.sa (K.C.)

[8] Laboratory of Analysis, Treatment and Valorization of Pollutants of the Environmental and Products, Faculty of Pharmacy, University of Monastir, Tunisia.

*Correspondence : Pr. Mejdi Snoussi (m.snoussi@uoh.edu.sa)

Supplementary Table S1. Details of virulence factors identified in the analyzed *S. aureus.*

Virulence mechanism	Related genes	MRSA	S1	S8	S9	S14	S20	S21	S22	S23
**Enzyme (15 Items)**										
Cysteine protease	sspB	SAR1021	orf00729	orf02372	orf02220	orf01632	orf00379	orf01616	orf00548	orf01529
Cysteine protease	sspC	SAR1020	orf00730	orf02371	orf02219	orf01631	orf00378	orf01617	orf00549	orf01530
Hyaluronate lyase	hysA	SAR2292	orf01573	orf02167	orf01632	orf01871	orf02310	orf00037	orf01270	orf01830
Lipase	geh	SAR0317	orf01915	orf00137	orf02309	orf00108	orf01968	orf01853	orf01920	orf00582
Lipase	lip	SAR2753	orf01366	orf00832	orf00265	orf01901	orf01335	orf01702	orf02435	orf01153
Serine V8 protease	sspA	SAR1022	orf00728	orf02373	orf00834	orf01633	orf00380	orf01615	orf00547	orf01528
Serine protease	splA	-	orf02216	orf02454	orf02221	-	orf01120	-	orf00381	orf02269
Serine protease	splB	-	orf02217	orf02455	orf01620	orf02487	orf01119	-	orf00380	orf02268
Serine protease	splC	SAR1906	orf02218	orf02456	orf01621	orf02488	orf01118	-	orf00379	orf02267
Serine protease	splD	SAR1905	orf02219	orf02457	orf01622	-	orf01117	-	-	orf02266
Serine protease	splE	SAR1902	orf02220	orf02551	orf01623	-	-	-	orf00377	orf02265
Serine protease	splF	SAR1900*	orf02221	orf02552	orf01624	orf02489	orf01114	-	orf00376	orf02264
Staphylocoagulase	coa	SAR0222	orf01274	orf00228	orf01625	orf00198	orf01555	orf01287	orf01535	orf00487
Staphylokinase	sak	SAR2039	orf01814	orf01688	orf00175	orf01021	orf02073	orf00943	orf00733	orf02079
Thermonuclease	nuc	SAR0847	orf00455	orf00736	orf01958	orf00466	orf00158	orf00468	orf00845	orf00925
			orf00886	orf01239	orf02427	orf00640	orf00650	orf00579	orf02225	orf01683
**Immune evasion (5 Items)**										
AdsA	adsA	SAR0023	orf01100	orf00765	orf00768	orf01673	orf01399	orf02264	orf01972	orf01217
CHIPS	chp	SAR2036	-	-	-	-	-	orf00941	orf00735	-
Capsule	--	SAR0151	orf01201	orf00291	orf00097	orf00254	orf01477	orf01215	orf01462	orf00415
		SAR0152	orf01202	orf00292	orf00098	orf00255	orf01478	orf01216	orf01463	orf00416
		SAR0153	orf01203	orf00293	orf00099	orf00256	orf01479	orf01217	orf01464	orf00417
		SAR0154	orf01205	orf00294	orf00100	orf00257	orf01480	orf01218	orf01465	orf00418
		SAR0155	orf01206	orf00296	orf00101	orf00258	orf01481	orf01219	orf01466	orf00419
		SAR0156	orf01207	orf00297	orf00102	orf00259	orf01482	orf01220	orf01467	orf00420
		SAR0157	orf01209	orf00298	orf00103	orf00260	orf01483	orf01221	orf01468	orf00421

		SAR0158	orf01210	orf00299	orf00104	orf00261	orf01484	orf01222	orf01469	orf00422
		SAR0159	orf01211	orf00300	orf00105	orf00262	orf01485	orf01223	orf01470	orf00423
		SAR0160	orf01212	orf00301	orf00106	orf00263	orf01486	orf01224	orf01471	orf00424
SCIN	scn	-	orf01816	orf01686	orf00107	orf01019	orf02075	orf00940	orf00736	orf02077
Sbi	sbi	-	orf02103	orf01074	orf00109	orf01193	orf01696	orf00268	orf01006	orf01939
**Secretion system (12 Items)**										
Type VII secretion system	esaA	SAR0280	orf01328	orf00172	orf00230	orf00142	orf01610	orf01343	orf01594	orf00545
Type VII secretion system	esaB	SAR0282	orf01330	orf00170	orf00232	-	orf01612	orf01345	orf01596	-
Type VII secretion system	esaD	-	orf01337	orf00163	orf00239	orf00133	orf01619	-	orf01603	orf00554
Type VII secretion system	esaE	-	orf01335	orf00165	orf00237	orf00135	orf01617	-	orf01601	orf00552
Type VII secretion system	esaG	SAR0293	orf01338	orf00155	orf00240	orf00126	orf01620	orf01834	orf01604	orf00555
		SAR0294	orf01341	orf00156	orf00244	orf00127	orf01623	orf01835	orf01607	orf00558
		-	orf01342	orf00157	orf00245	orf00128	orf01624	-	orf01610	orf00559
		-	orf01343	orf00158	orf00246	orf00129	orf01625	-	orf01611	orf00560
Type VII secretion system	essA	SAR0281	orf01329	orf00171	orf00231	orf00141	orf01611	orf01344	orf01595	orf00546
Type VII secretion system	essB	SAR0283	orf01331	orf00169	orf00233	orf00139	orf01613	orf01346	orf01597	orf00548
Type VII secretion system	essC	SAR0284	orf01332	orf00168	orf00234	orf00138	orf01614	orf01347	orf01598	orf00549
Type VII secretion system	esxA	SAR0279	orf01327	orf00173	orf00229	orf00143	orf01609	orf01342	orf01593	orf00544
Type VII secretion system	esxB	-	orf01334	orf00166	orf00236	orf00136	orf01616	-	orf01600	orf00551
Type VII secretion system	esxC	-	orf01333	orf00167	orf00235	orf00137	orf01615	-	orf01599	orf00550
Type VII secretion system	esxD	-	orf01336	orf00164	orf00238	orf00134	orf01618	-	orf01602	orf00553
**Toxin (74 Items)**										
Alpha hemolysin	hly/hla	SAR1136*	orf00617	orf01436	orf01506	orf02561	orf00489	orf01210	orf00438	orf00758
Beta hemolysin	hlb	SAR2031*	orf01765	orf01746	orf02411	orf01079	orf02027	orf00997	orf02080	orf02243
Delta hemolysin	hld	SAR2122	orf01750	orf01781	orf02376	-	orf02012	orf01030	orf02067	orf02230
Enterotoxin A	sea	SAR2043	orf01811	-	-	-	orf02070	-	-	orf02082
Enterotoxin B	seb	-	orf00896	-	-	orf00044	-	-	-	-

Enterotoxin C	sec	-	-	-	-	-	-	-	-	-
Enterotoxin D	sed	-	-	-	-	-	-	-	-	-
Enterotoxin E	see	-	-	-	-	-	-	-	-	-
Enterotoxin G	seg	SAR1916	-	-	-	orf02473	-	orf02533	-	-
Enterotoxin H	seh	-	orf01128	-	-	-	orf01404	-	-	-
Enterotoxin I	sei	SAR1919	-	-	-	orf02522	-	orf02530	-	-
Enterotoxin J	sej	-	-	-	-	-	-		-	-
Enterotoxin Yent1	yent1	-	-	-	-	-	-		-	-
Enterotoxin Yent2	yent2	-	-	-	-	-	-		-	-
Enterotoxin-like K	selk	-	orf01767	-	-	-	orf02029	-	-	-
Enterotoxin-like L	sell	-	-	-	-	-	-		-	-
Enterotoxin-like M	selm	SAR1920	-	-	-	orf02521	-	orf02529	-	-
Enterotoxin-like N	seln	SAR1917	-	-	-	orf02524	-	orf02532	-	-
Enterotoxin-like O	selo	SAR1921	-	-	-	orf02520	-	orf02541	-	-
Enterotoxin-like P	selp	-	-	-	-	-	-	-	-	-
Enterotoxin-like Q	selq	-	orf01768	-	-	-	orf02030	-	-	-
Enterotoxin-like R	selr	-	-	-	-	-		-	-	-
Enterotoxin-like U	selu	-	-	-	-	orf02523		orf02531	-	-
Exfoliative toxin type A	eta	-	-	-	-	-	-	-	-	-
Exfoliative toxin type B	etb	-	-	-	-	-	-	-	-	-
Exfoliative toxin type C	etc	-	-	-	-	-	-	-	-	-
Exfoliative toxin type D	etd	-	-	-	-	-	-	-	-	-
Exotoxin	set10	-	-	-	-	-	-	orf02141	-	-
Exotoxin	set11	-	-	-	-	-	-	-	-	-
Exotoxin	set12	-	-	-	-	-	-	-	-	-
Exotoxin	set13	-	-	-	-	-	-	orf02139	-	orf00674
Exotoxin	set14	-	-	-	-	-	-	-	-	-
Exotoxin	set15	-	-	-	-	orf01972	-	orf02135	-	orf02405
Exotoxin	set16	-	orf01997	orf00059	orf00344	-	orf01862	orf02145	orf01845	orf00665
Exotoxin	set17	-	orf01998	-	-	-	orf01861	-	-	-

Exotoxin	set18	-	orf01999	orf00057	orf00346	orf00008	orf01860	-	-	orf00667
Exotoxin	set19	-	orf02001	orf00055	orf00347	orf00007	orf01858	-	-	-
Exotoxin	set1	SAR0428	-	-	-	-	-	orf02140	orf01839	-
Exotoxin	set20	-	-	-	-	-	-	-	-	-
Exotoxin	set21	-	orf02003	-	-	orf00005	orf01856	-	-	-
Exotoxin	set22	-	orf02004	orf00053	orf00349	orf00004	orf01855	-	-	orf00672
Exotoxin	set23	-	orf02005	-	-	-	orf01854	-	-	-
Exotoxin	set24	-	orf02006	-	-	-	-	-	-	-
Exotoxin	set25	-	orf02007	orf00050	orf00352	orf00001	orf01853	-	-	orf00675
Exotoxin	set26	-	orf02010	orf00047	orf00355	-	orf01850	-	orf01834	-
Exotoxin	set2	SAR0425	-	-	-	-	-	orf02143	orf01842	-
Exotoxin	set30	-	-	-	-	orf00010	-	-	-	-
Exotoxin	set31	-	-	-	-	orf00009	-	-	-	-
Exotoxin	set32	-	-	-	-	-	-	-	-	-
Exotoxin	set33	-	-	-	-	-	-	-	-	-
Exotoxin	set34	-	orf02002	orf00054	orf00348	orf00006	orf01857	-	-	orf00671
Exotoxin	set35	-	-	-	-	-	-	-	-	-
Exotoxin	set36	-	-	-	-	-	-	-	-	-
Exotoxin	set37	-	-	orf00052	orf00350	orf00003	-	-	-	orf00673
Exotoxin	set38	-	-	orf00051	orf00351	orf00002	-	-	-	-
Exotoxin	set39	-	-	-	-	-	-	-	-	-
Exotoxin	set3	SAR0427	-	-	-	-	-	-	orf01840	-
Exotoxin	set40	-	-	-	-	-	-	-	-	-
Exotoxin	set4	SAR0431	-	-	-	-	-	orf02138	orf01837	-
Exotoxin	set5	SAR0429	-	-	-	-	-	-	orf01838	-
Exotoxin	set6	-	-	-	-	-	-	-	-	-
Exotoxin	set7	-	-	orf00058	orf00345	-	-	orf02144	orf01844	orf00666
Exotoxin	set8	-	-	-	-	-	-	-	-	-
Exotoxin	set9	-	-	-	-	-	-	-	-	-
Gamma hemolysin	hlgA	SAR2509	orf02104	orf01073	orf01074	orf01194	orf01131	orf00270	orf00388	orf01938

			orf02204	orf02448	orf01613	orf02479	orf01695	-	orf01007	orf02280
Gamma hemolysin	hlgB	SAR2511	orf02106	orf01071	orf01072	orf01196	orf01693	orf00272	orf01009	orf01936
Gamma hemolysin	hlgC	SAR2510	orf02105	orf01072	orf01073	orf01195	orf01694	orf00271	orf01008	orf01937
Leukocidin M	lukF-like	-	-	-	-	-	-	-	-	-
Leukocidin M	lukM	-	-	-	-	-	-	-	-	-
Leukotoxin D	lukD	SAS1748	orf02205	orf02449	orf01614	orf02480	orf01130	-	orf00387	orf02279
Leukotoxin E	lukE	SAS1749	-	-	-	-	-	-	-	-
Panton-Valentine leukocidin	lukF-PV	-	-	-	-	orf02119	-	-	-	-
Panton-Valentine leukocidin	lukS-PV	-	-	-	-	orf02120	-	-	-	-
Toxic shock syndrome toxin	tsst	-	-	-	-	-	-	orf01006	-	-

Supplementary Table S2. Predicted virulence factors in the whole genome of the studied *S. aureus*.

Virulence mechanism	Related genes	MRSA252	S1	S8	S9	S14	S20	S21	S22	S23
**Enzyme (8 Items)**										
Staphylocoagulase	1	+	+	+	+	+	+	+	+	+
Serine protease	6	3 / 1	+	+	+	3	5	-	5	+
Cysteine protease	2	+	+	+	+	+	+	+	+	+
Thermonuclease	1	+	+	+	+	+	+	+	+	+
Hyaluronate lyase	1	+	+	+	+	+	+	+	+	+
Lipase	2	+	+	+	+	+	+	+	+	+
Serine V8 protease	1	+	+	+	+	+	+	+	+	+
Staphylokinase	1	+	+	+	+	+	+	+	+	+
**Immune evasion (5 Items)**										
SCIN	1	+	+	+	+	+	+	+	+	+
Sbi	1	+	+	+	+	+	+	+	+	+
CHIPS	1	+	-	-	-	-	-	+	+	-
AdsA	1	+	+	+	+	+	+	+	+	+
Capsule	1	+	+	+	+	+	+	+	+	+
**Secretion system (1 Item)**										
Type VII secretion system	12	7	+	+	+	11	+	7	+	11
**Toxin (34 Items)**										
Panton-Valentine leukocidin	2	-	-	-	-	+	-	-	-	-
Toxic shock syndrome toxin	1	-	-	-	-	-	-	+	-	-
Exotoxin	37	5	11	10	10	11	10	8	8	9
Enterotoxin Yent2	1	-	-	-	-	-	-	-	-	-
Enterotoxin Yent1	1	-	-	-	-	-	-	-	-	-
Enterotoxin-like U	1	+	-	-	-	+	-	+	-	-
Enterotoxin-like Q	1	-	+	-	-	-	+	-	-	-
Enterotoxin-like P	1	-	-	-	-	-	-	-	-	-
Enterotoxin-like O	1	+	-	-	-	+	-	+	-	-

Enterotoxin-like N	1	+	-	-	-	+	-	+	-	-
Enterotoxin-like M	1	+	-	-	-	+	-	+	-	-
Enterotoxin-like L	1	-	-	-	-	-	-	-	-	-
Enterotoxin-like K	1	-	+	-	-	-	+	-	-	-
Enterotoxin I	1	+	-	-	-	+	-	+	-	-
Enterotoxin H	1	-	+	-	-	-	+	-	-	-
Enterotoxin G	1	+	-	-	-	+	-	+	-	-
Enterotoxin C	1	-	-	-	-	-	-	-	-	-
Enterotoxin B	1	-	+	-	-	+	-	-	-	-
Enterotoxin A	1	+	+	-	-	-	+	-	-	+
Enterotoxin D	1	-	-	-	-	-	-	-	-	-
Enterotoxin E	1	-	-	-	-	-	-	-	-	-
Enterotoxin J	1	-	-	-	-	-	-	-	-	-
Exfoliative toxin type B	1	-	-	-	-	-	-	-	-	-
Exfoliative toxin type C	1	-	-	-	-	-	-	-	-	-
Exfoliative toxin type D	1	-	-	-	-	-	-	-	-	-
Exfoliative toxin type A	1	-	-	-	-	-	-	-	-	-
Enterotoxin-like R	1	-	-	-	-	-	-	-	-	-
Delta hemolysin	1	+	+	+	+	-	+	+	+	+
Gamma hemolysin	3	+	+	+	+	+	+	+	+	+
Beta hemolysin	1	-	+	+	+	+	+	+	+	+
Alpha hemolysin	1	-	+	+	+	+	+	+	+	+
Leukocidin M	2	-	-	-	-	-	-	-	-	-
Leukotoxin E	1	+	-	-	-	-	-	-	-	-
Leukotoxin D	1	+	+	+	+	+	+	-	+	+
